# Anesthetic and surgical considerations of giant pericardial cyst: Case report and literature review

**DOI:** 10.1016/j.amsu.2020.05.038

**Published:** 2020-06-03

**Authors:** Rami Alqassieh, Mahmoud Al-Balas, Hamzeh Al-Balas

**Affiliations:** Department of General and Special Surgery, Faculty of Medicine, Hashemite University, Jordan

**Keywords:** Pericardial cyst, Mediastinal mass, Bronchogenic cyst, Case report

## Abstract

**Introduction:**

Pericardial cysts are considered as a rare congenital abnormality, mostly found incidentally. The estimated incidence of pericardial cyst is 1:100,000 and represent approximately 6% of all mediastinal masses. Patients can present with symptoms similar to acute chest pain or right-sided heart failure or can be asymptomatic.

**Presentation:**

A 46-year-old female who is known to have hypertension and hypothyroidism presented to the breast clinic with left breast mass that was proved by core needle biopsy as proliferative breast lesion. During the preoperative assessment, the patient reported progressive shortness of breath and cough over the last two years and bilateral lower limb edema. Her preoperative chest X-ray showed a well-defined oval like opacification at the right cardiophrenic angle that was proved by chest computed tomography imaging as a cystic mass od most likely a pericardial origin. A huge pericardial cyst originating from the right diaphragmatic surface was excised through a mini-sternotomy incision with smooth postoperative recovery. The patient-reported significant improvement in her symptoms and her lifestyle during her follow up.

**Discussion:**

Pericardial cysts represent 6%–7% of all mediastinal masses with an estimated incidence of 1:100,000. About 70% of pericardial cysts originate at the right cardiophrenic angle and less frequently at the left cardiophrenic angle, they are usually suspected when the chest x-ray shows an enlarged contour of the right heart border. Mediastinal cysts have many differential diagnoses and the preoperative decision might be challenging in many cases. Pericardial cysts appear as oval, thin-walled homogeneous masses on cardiac computed tomography. The choice between surgical intervention and conservative follow up is related mainly to the size and symptoms that are induced by the cyst.

**Conclusion:**

As pericardial cysts are rarely diagnosed pathology, a high index of suspicion is essential for diagnosis. Surgical resection is indicated when they are huge, enlarging in size or symptomatic. Morbidity and mortality risks following pericardial cyst excision are very low.

## Highlights

• Pericardial cysts are rare congenital abnormalities.
• Symptoms of right sided heart failure or unexplained chronic cough and dyspnea may suggest pericardial cyst as an underlying pathology.
• The morbidity and mortality risks following pericardial cyst excision are very low.

## 1 Introduction

Pericardial cysts are considered as a congenital abnormality, mostly present with chest pain, shortness of breath, and cough [1], in few reported cases, huge pericardial cysts can also be discovered incidentally in asymptomatic patients [[2], [3], [4], [5]]. Pericardial cysts represent 6%–7% of all mediastinal masses with an estimated incidence of 1:100,000 [6]. In some cases, pericardial cysts can be discovered incidentally on x-ray but CT or MRI chest is the preferable method for diagnosis. They are usually smooth unilocular cysts with a diameter of less than 3 cm. Most authorities recommend the removal of the cyst if the diagnosis is in doubt or if the patient complains of severe symptoms. Her in; we present a case of a 46-year-old lady with a huge pericardial cyst that was discovered incidentally during her preoperative assessment for breast surgery, and its surgical anesthetic considerations. This case is reported in concordance with the SCARE 2018 criteria [7].

### 1.1 Case report

A 46-year-old housewife Arabic lady, with a known history of hypothyroidism and hypertension for the last 5 years, presented to our breast surgery clinic complaining of a left breast mass that was diagnosed during her mammographic screening. This breast mass was proved by a core biopsy as a proliferative breast lesion.

During her clinic visit, the patient reported shortness of breath and dry cough for two years duration. Her dyspnea was progressive, and for the last 6 months she started to complain from orthopnea. The patient didn't have palpitations, syncopal attacks, or hemoptysis. There was no history of trauma, previous chest infection, or cardiothoracic operation. She was followed by her physician during this period where he related her symptoms for hypothyroidism, no further evaluation or imaging was performed for her. Regarding her medications, she is receiving l-thyroxine tablets (100 μg) and metoprolol 100mg daily for the last 5 years. She had a previous history of appendectomy and unremarkable family history; she is nonsmoker with no known allergic history.

On examination, vitals were stable, with normal regular heart rhythm and normal heart sounds. The rest of her examination was unremarkable except for grade 2 bilateral lower limb edema. Her ECG showed a normal heart rate with sinus rhythm.

**Fig. 1:**
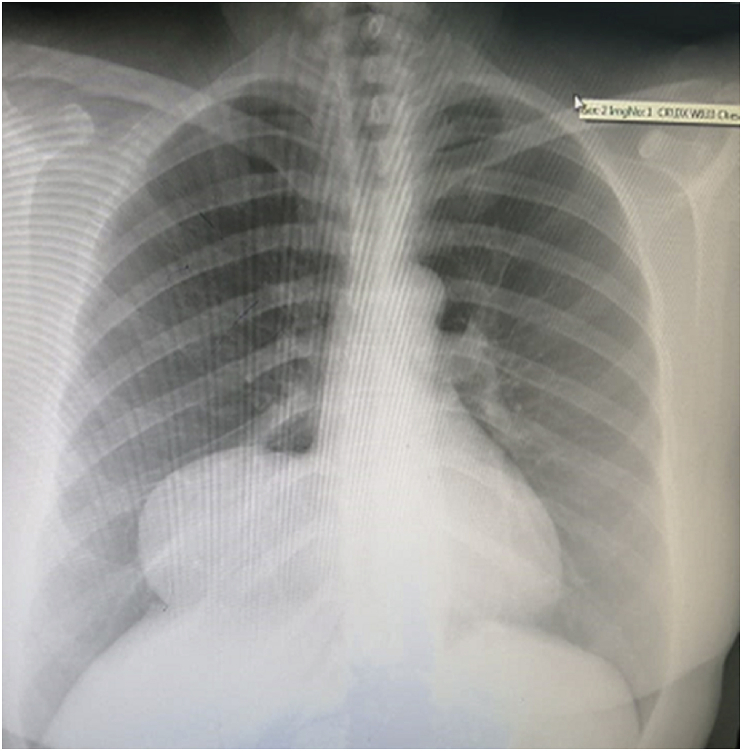
A chest x-ray shows a well-defined oval like opacification at the right cardiophrenic angle.

**Fig. 2:**
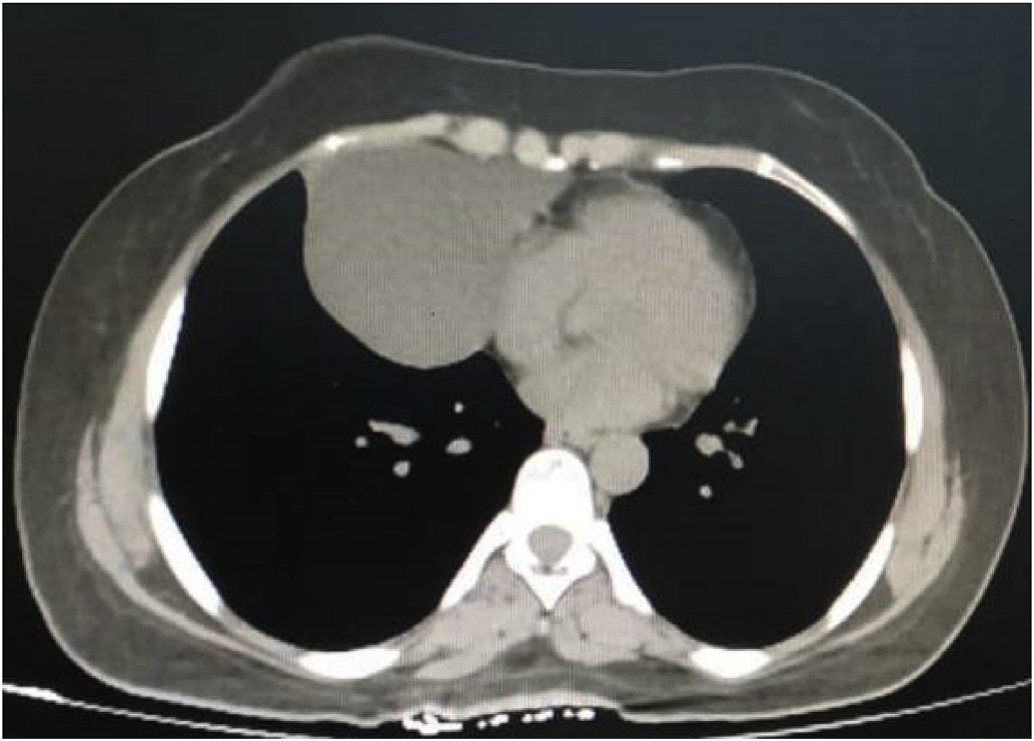

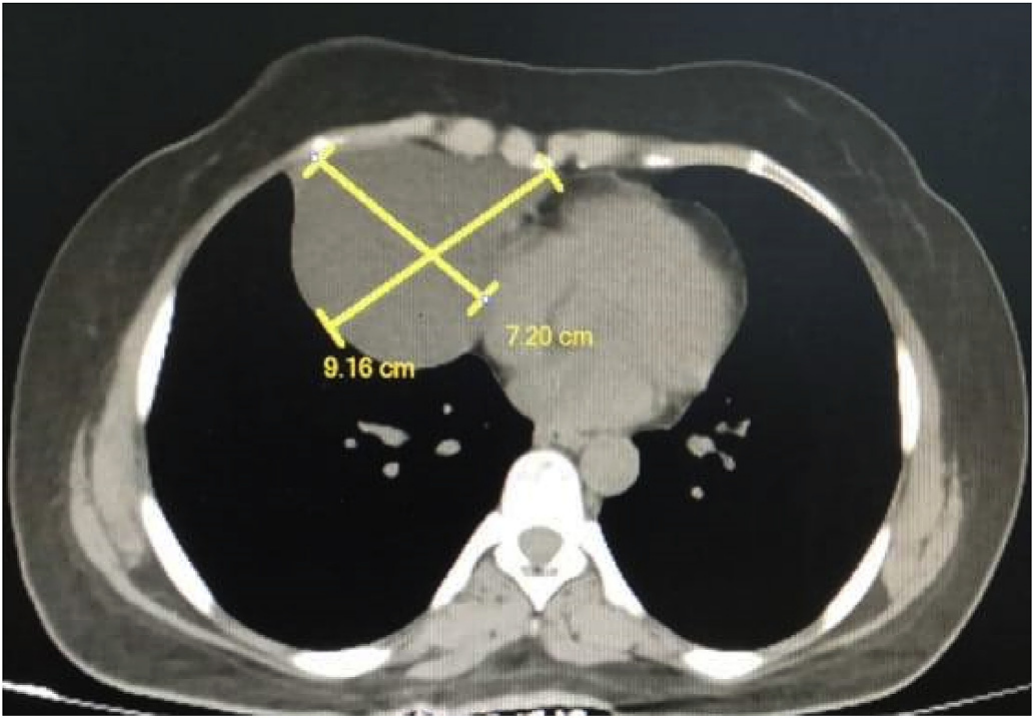
A chest CT scan with contrast shows homogeneous mass occupying the right lower lung lobe Figure 2B: a chest CT scan with contrast shows homogeneous mass measuring 7.2 × 9.16 cm occupying the right lower lung lobe.

As a preoperative assessment, a chest x-ray showed a well-defined oval like opacification at the right cardiophrenic angle [Fig. 1]. After that, a chest CT scan with contrast performed and a homogeneous mass measuring 7.2 × 9.1 cm occupying the right lower lung lobe was identified, it was consistent with the pericardial cyst although bronchogenic cyst could not be ruled out [Fig. 2A and B].

The patient was referred for the cardiac surgery department for further evaluation. Cardiac echography demonstrated a large pericardial cyst 7.5 × 7 cm compressed to the right atrium, grade 1 tricuspid valve regurgitation with a cardiac ejection fraction of 60%, the rest of the echocardiography was normal. Complete blood count, liver function tests, serum creatinine, and electrolyte levels performed, all result within the normal range and values. ELISA scan was performed to rule out the possibility of a hydatid cyst. Urine analysis showed no evidence of microscopic hematuria and abdominal ultrasound showed normal kidneys eliminating the possibility of polycystic kidney disease.

Upon on available findings, the most likely diagnosis was a pericardial cyst and the decision was to excise the cyst using a mini-sternotomy incision.

On the day of surgery, patient vitals were normal (blood pressure of 126/79 mmHg, heart rate of 88 beats per minute, respiratory rate of 16 per minute, blood oxygen saturation of 97% on room air, and temperature of 36.9∘ C). The standard American Society of Anesthesiologists monitors were placed on the patient; two large-bore intravenous catheters; left radial arterial line and central venous catheter in the right internal jugular were inserted. The patient was hydrated using ringer lactate.

The patient was pre oxygenated and general anesthesia was induced by slow intravenous administration of etomidate 0.2 mg/kg and fentanyl 1 mic/kg. Neuromuscular blockade was achieved by intravenous administration of succinylcholine 1.5 mg/kg. Ephedrine 0.1 mg/kg was administered following induction to minimize the hemodynamic effects of the inductive agents and positive pressure ventilation. In a supine position, the patient was intubated using a 7.5 mm endotracheal tube. Anesthesia was maintained with oxygen (fraction of inspired oxygen of 0.6) and sevoflurane (1 minimum alveolar concentration). Neuromuscular blockade was induced with rocuronium 0.6 mg/kg.

**Fig. 3:**
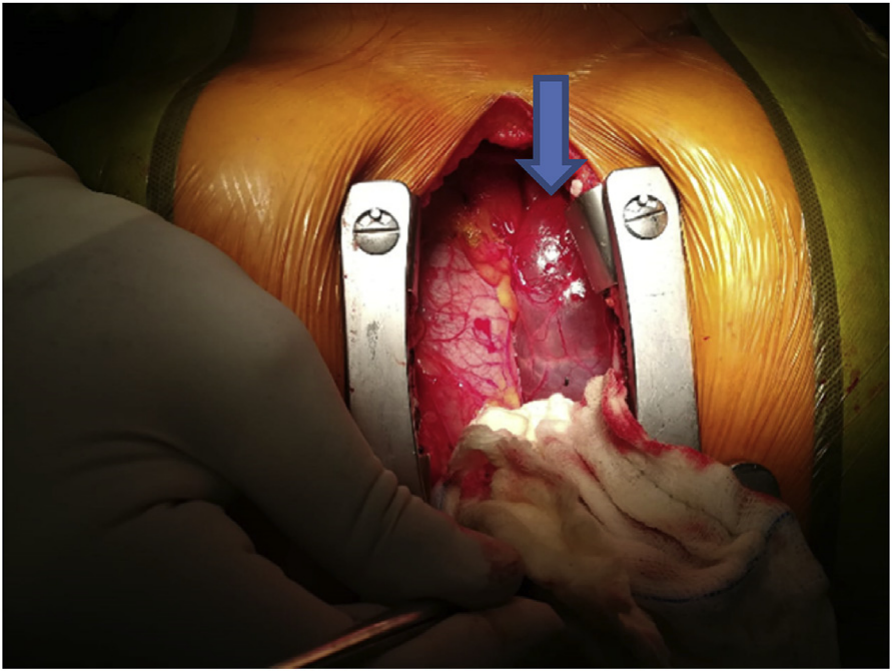

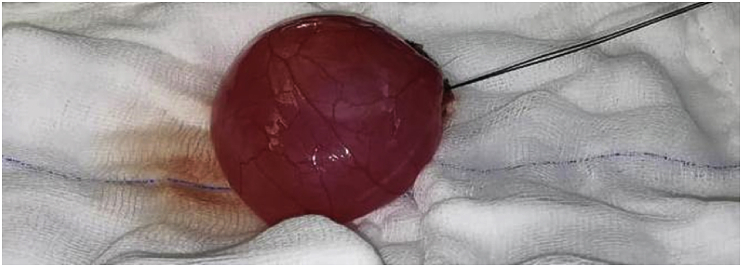
A mini-sternotomy with a huge pericardial cyst with clear content Figure 3B: pericardial cyst after excision.

The procedure was performed by our consultant cardiothoracic surgeon at Prince Hamza Hospital cardiac unit. Using a mini-sternotomy incision, a huge pericardial cyst measuring approximately 8 × 8 cm arising from the diaphragmatic surface of the right pericardium was identified and excised smoothly with identification and preservation of the phrenic nerve [Fig. 3A and B]. During the pericardial cyst excision, patient vitals remained stable and the patient was extubated successfully without any complications.

The patient had smooth postoperative recovery and her symptoms improved dramatically, she was discharged home 48 hours later on analgesics. Histopathologic analysis of the specimen demonstrated a fibrous membranous tissue with mesothelial lining. The cystic fluid was clear and fluid analysis was unremarkable. Her first follow up visit was arranged one week later where she reported a complete resolution of her symptoms; her new CXR and ECHO were unremarkable. The patient was advised to avoid heavy lifting for 6 weeks and self-wound care. She was followed on 3 months interval, and she remained symptom-free during her 12-month follow-up.

## 2 Discussion

Pericardial cysts represent 6%–7% of all mediastinal masses with an estimated incidence of 1:100,000 [6]. Anatomically, the cyst has a single layer of mesothelial cells composed of connective tissue with grossly clear fluid content [8]. They are usually smooth unilocular cysts with a diameter of less than 3 cm [9].

Approximately, 70% of pericardial cysts originate at the right cardiophrenic angle followed by the left cardiophrenic angle (22%). Both posterior and anterior superior mediastinum accounts for the remaining 8% of cases [10,11]. A pericardial cyst is usually suspected as in this case when the chest x-ray shows an enlarged contour of the right heart border.

Radiologically, Mediastinal cysts have many differential diagnoses as a bronchogenic cyst, esophageal duplication cyst, neurenteric cyst, thymic cysts, and other types of cysts [12]. Pericardial cysts appear as oval, thin-walled, and well-defined homogeneous masses on cardiac computed tomography [13]. The choice to keep observational management with serial echocardiography or surgical one depends on the size and symptoms that are induced by the cyst. Management options for mediastinal cyst vary from less invasive ones like percutaneous aspiration under ultrasound or echocardiography to more invasive procedures like thoracotomy, video-assisted thoracoscopy, or sternotomy [14].

As mentioned previously, some cases can be followed with no need for any intervention. If this is the case, complications such as rupture of the cyst in case of lost follow up or compression effect to the heart, lung, main bronchus, superior vena cava, or any mediastinal structures are expected [15,16]. It also may lead to hemorrhage, infection, or tamponade [17,18].

The decision to proceed with any surgical approach depends on the surgeon's experience, the general condition of the patient, size, and location of the cyst. In our opinion, the surgical option which consists of cyst excision is the preferable option for the management regardless of the surgical technique. A review of literature for reported and published huge or large pericardial cyst is summarized in Table 1.

**Table 1:** Summary of published giant/large pericardial cyst case reports.

Reference	Publication year	Age (years)	Gender	Size (cm)	Presentation	Location	treatment
Gharedaghi et al. [2]	2019	57	Male	9	Asymptomatic	Right side	Video-assisted thoracoscopic excision
Makar et al. [23]	2018	43	Female	9 × 5	Progressive cough and chest tightness	Right side	Video-assisted thoracoscopic excision Video-assisted thoracoscopic excision
Akbayrak et al. [3]	2016	48	Male	27 × 5	Asymptomatic	Right side	Surgical excision with median sternotomy
Hekmat et al. [24]	2016	24	Male	13 × 8	Dyspnea and cough	Right side	Surgical excision with median sternotomy
Simsek et al. [25]	2014	28	Male	6.4 × 9	Dry cough and pleuritic chest pain	Right side	Conservative
Hamad et al. [26]	2013	30	Male	11.2 × 7.4	Palpitation and chest pain	Right side	Surgical excision
Celik et al. [27]	2012	54	Male	6.5 × 4.7	ST elevation myocardial infarction	Left side	Cardiac bypass surgery and surgical excision
Forouzandeh et al. [28]	2012	71	Female	8 × 5	Cough, dyspnea, fever	Right side	Video-assisted thoracoscopic excision
Thanneer et al. [29]	2011	22	Female	21.5 × 14.2	Syncope	Posterior mediastinum (behind the heart)	Needle aspiration
Kumar et al. [30]	2011	5	Male	10 × 9.5	Cough, chest pain and fever	Right side	Median sternotomy cyst excision
Kaklikkaya I [31]	2011	39	Male	22 × 15	Pleuritic chest pain	Left side	Left thoracotomy cyst excision
Matono et al. [4]	2010	38	Male	12 × 10	Asymptomatic	Right side	Video-assisted thoracoscopic excision
Neizel et al. [32]	2010	59	Female	5 × 5	Pre-syncopal and atrial flutter	Behind the heart	Surgical excision
Pereira et al. [33]	2008	73	Female	14 × 10	Chest pain	Right, anterior and left side of the heart	Not operated
Nina et al. [34]	2007	44	Female	13 × 9.5′	Exertional dyspnea, dry cough and chest pain	Right side	Right thoracotomy cyst excision
Dernellis et al. [5]	2001	27	Female	15.6 × 12.2	Asymptomatic	Right side	Surgical excision

A giant pericardial cyst may result in significant physiologic alterations, so anesthesiologists need to understand this situation. The right-sided pericardial cyst may cause significant compression of the right atrium, right main bronchus, and superior vena cava. Because of that, giant right pericardial cysts may lead to a reduction of preload and cardiac output [19]. In such a situation, significant fluid resuscitation before the induction of anesthesia is mandatory. Patients with features of SVC compression are recommended to have their intravenous lines in the lower extremities [20] and to place patients preferably in a sitting or Semi-recumbent position to avoid the possibility of an increase in the intracranial pressure.

In patients with symptoms suggestive of airway compression or during patient positioning, the induction of general anesthesia can exacerbate their situation by decreasing the negative intrathoracic pressure and relaxing the bronchial smooth muscles. Neuromuscular blockage or positive pressure ventilation can further exacerbate airway compression. To avoid such a situation, inhalational induction or awake fiberoptic endotracheal intubation can be considered [20,21].

Regarding the hemodynamic stability, it is preferred to use drugs with minimal hemodynamic or with sympathomimetic effects such as Ketamine and etomidate for induction of anesthesia. Ephedrine can also be used during induction to increase vasoconstriction and cardiac output [2,22].

In our literature review, the morbidity and mortality risks following pericardial cyst excision are very low and complications with tracheal extubation in such cases were not reported.

## Ethical approval

Ethical approval is obtained from the intuitional review board at Hashemite University

## Funding source

This research did not receive any funding from any resource.

## Author contribution

Study design, writing, and the final approval of the manuscript: Dr Rami Alqassieh.

Study design, Literature review and Data collection: Dr. Hamzeh Al-Balas.

Literature review, patient follow up and manuscript revision: Dr. Mahmoud Al-Balas.

## Registration of research studies

Non applicable.

## Guarantor

Dr. Mahmoud Al-Balas.

## Patient perspective

Follow up in cardiology clinic if any new symptoms develop in future.

## Informed consent

Written informed consent was obtained from the patient for publication of this case report and accompanying images. A copy of the written consent is available for review by the Editor-in-Chief of this journal on request.

## Declaration of competing interest

There is no conflict of interest.
